# Efficient display of active lipase LipB52 with a *Pichia pastoris *cell surface display system and comparison with the LipB52 displayed on *Saccharomyces cerevisiae *cell surface

**DOI:** 10.1186/1472-6750-8-4

**Published:** 2008-01-28

**Authors:** Zhengbing Jiang, Bei Gao, Ren Ren, Xingyi Tao, Yushu Ma, Dongzhi Wei

**Affiliations:** 1 State Key Laboratory of Bioreactor Engineering, Newworld Institute of Biotechnology, East China University of Science and Technology, Shanghai 200237, P. R. China; 2 College of Life Science, Hubei University, Wuhan 430062, P. R. China

## Abstract

**Background:**

For industrial bioconversion processes, the utilization of surface-displayed lipase in the form of whole-cell biocatalysts is more advantageous, because the enzymes are displayed on the cell surface spontaneously, regarded as immobilized enzymes.

**Results:**

Two *Pichia pastoris *cell surface display vectors based on the flocculation functional domain of FLO with its own secretion signal sequence or the α-factor secretion signal sequence were constructed respectively. The lipase gene *lipB52 *fused with the *FLO *gene was successfully transformed into *Pichia pastoris *KM71. The lipase LipB52 was expressed under the control of the *AOX1 *promoter and displayed on *Pichia pastoris *KM71 cell surface with the two *Pichia pastoris *cell surface display vectors. Localization of the displayed LipB52 on the cell surface was confirmed by the confocal laser scanning microscopy (CLSM). The LipB52 displayed on the *Pichia pastoris *cell surface exhibited activity toward *p*-nitrophenol ester with carbon chain length ranging from C_10 _to C_18_, and the optimum substrate was *p*-nitrophenol-caprate (C_10_), which was consistent with it displayed on the *Saccharomyces cerevisiae *EBY100 cell surface. The hydrolysis activity of lipase LipB52 displayed on *Pichia pastoris *KM71-pLHJ047 and KM71-pLHJ048 cell surface reached 94 and 91 U/g dry cell, respectively. The optimum temperature of the displayed lipases was 40°C at pH8.0, they retained over 90% activity after incubation at 60°C for 2 hours at pH 7.0, and still retained 85% activity after incubation for 3 hours.

**Conclusion:**

The LipB52 displayed on the *Pichia pastoris *cell surface exhibited better stability than the lipase LipB52 displayed on *Saccharomyces cerevisiae *cell surface. The displayed lipases exhibited similar transesterification activity. But the *Pichia pastoris *dry cell weight per liter (DCW/L) ferment culture was about 5 times than *Saccharomyces cerevisiae*, the lipase displayed on *Pichia pastoris *are more suitable for whole-cell biocatalysts than that displayed on *Saccharomyces cerevisiae *cell surface.

## Background

Lipases (E.C.3.1.1.3) are esterases able to hydrolyze water-insoluble esters, which have a wide range of potential industrial applications [1-3]. *Pseudomonas *lipases display special biochemical characteristics differing from those produced by other microorganisms [1,4-6]. The lipase LipB52 encoded by *lipB52 *(GenBank accession number AY623009), isolated from *Pseudomonas fluorescens *B52, displayed high level enantioselectivity with (*R*)-*tert*-leucinate [7]. For industrial bioconversion processes, the utilization of surface-displayed lipase in the form of whole-cell biocatalysts is more advantageous, because the enzymes are displayed on the cell surface spontaneously, regarded as immobilized enzyme, and can be separated easily. With the recent development of cell-surface display technology, many active enzymes can be genetically immobilized on *Saccharomyces cerevisiae *cell surface [8,9], while displaying active enzymes on *Pichia pastoris *cell surface was rarely reported[10,11]. In fact, the *P. pastoris *expression system has gained acceptance as an important host organism[12], and is common for high level expression of foreign proteins.

In this paper, we have the lipase LipB52 displayed on *Pichia pastoris *KM71 cell surface with a *Pichia pastoris *cell surface display system based on the *FLO *gene encoding a lectin-like cell-wall protein (FLO) from *Saccharomyces cerevisiae *[13]. FLO is composed of several domains: the secretion signal domain, the flocculation functional domain, the glycosyl phosphatidylinositol (GPI) anchor attachment signal domain and the membraneanchoring domain [14-16]. The FLO flocculation functional domain, thought to be located near the N-terminus, recognizes and adheres noncovalently to cell-wall components such as α-mannan carbohydrates, causing reversible aggregation of cells into flocs [13,14,16]. The 5'-terminus of lipase gene *lipB52 *was fused to the 3'-terminus of the *FLO *gene with the secretion signal sequence of *FLO *or the α-factor secretion signal sequence, and the fused gene was transformed into a heterologous fungal host, *Pichia pastoris *KM71. The lipase LipB52 was expressed under the control of the *AOX1 *promoter and displayed on *Pichia pastoris *KM71 cell surface. Localization of the expressed lipase LipB52 on the cell surface was confirmed by the confocal laser scanning microscopy (CLSM). Some biochemical characteristics of the lipase LipB52 displayed on *Pichia pastoris *KM71 cell surface were also analysed and compared with the LipB52 displayed on *Saccharomyces cerevisiae *EBY100-pLHJ026 cell surface.

## Results

### Construction of the recombinant plasmids of *FLO-lipB52 *and expression of fusion proteins

**Figure 1:**
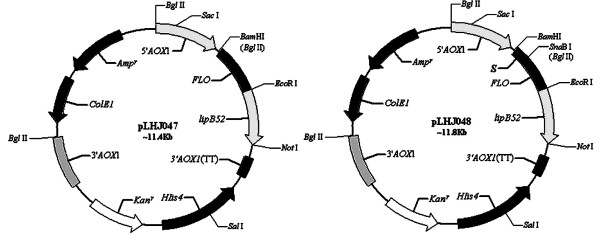
**The structure of the expression plasmid pLHJ047 and p LHJ048**. 5'*AOX*1, alcohol oxidase 1 promoter; *S*, α-factor signal sequence for secretion in *Pichia*; *FLO*, *FLO *gene coding for FLO protein from *Saccharomyces cerevisiae*; *lipB52*, lipase gene *lipB52 *cloned in frame and downstream of the *FLO *sequence; 3'*AOX*1(TT), transcriptional terminator from *pichia pastoris AOX1*gene; *HIS4*, *pichia *wild-type gene coding for histidinol dehydrogenase and used to complement *Pichia *his4^- ^strains; *kan*^*r*^, Kanamycin resistance gene from Tn*903 *which confers resistance to G418 in *Pichia*; 3'*AOX1*, Sequences from the *AOX1 *gene that are further 3' to the TT sequences; *CoE1*, *E. coli *origin of replication; *Amp*^*r*^, Ampicillin resistance gene.

The recombinant plasmids pLHJ047 and pLHJ048 (Fig. 1) were constructed as described in Methods. The *FLO *sequence was cloned in the frame and downstream of the α-factor secretion signal sequence in pLHJ048, while the secretion signal sequence in pLHJ047 was the one from *FLO*.

**Figure 2:**
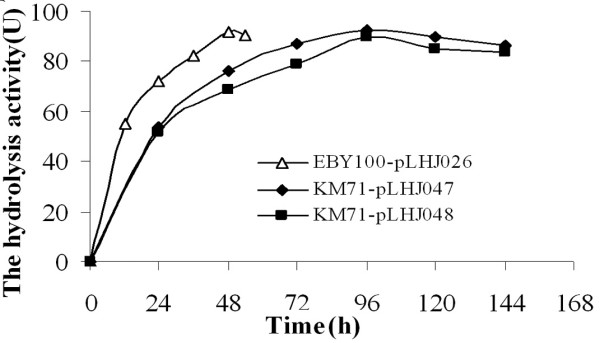
**The curve of displayed lipase production**. The lipase activity produced by *pichia pastoris *KM71-pLHJ047 reached its maximum 92 U/g dry cell after induced for 96 h; The lipase activity produced by *pichia pastoris *KM71-pLHJ048 reached its maximum 89 U/g dry cell after induced for 96 h; The lipase activity produced by *Saccharomyces cerevisiae *EBY100-pLHJ026 reached its maximum 92 U/g dry cell after induced for 48 h. Assayed under the same condition: *p*-nitrophenol-caprate used as substrate, assayed at 37°C, pH8.0.

The recombinant plasmids linearized with *Sal *I were electroporated into *Pichia pastoris *KM71 as described in Methods. The lipase gene was expressed under the control of the *AOX1 *promoter and the displayed lipase activity were detected by BMMY medium plates supplemented with 1% olive oil and 0.002% rhodamine B, the expression pattern can be determined by the fluorescent halo around them. The single transformant colony with lipase activity was inoculated and named as KM71-pLHJ047 and KM71-pLHJ048, respectively. By using the total DNA of KM71-pLHJ047 and KM71-pLHJ048 as the template, a DNA fragment with the same size as lipase gene *lipB52 *was obtained with primers LipB52Pf-*Eco*R I and LipB52Pr-*Not *I by PCR amplification (total DNA of *Pichia pastoris *KM71 transfected with the plasmid pPIC9K was used as the negative control template), which convinced that the KM71-pLHJ047 and KM71-pLHJ048 were recombinant *Pichia pastoris *with lipase gene *lipB52*. The lipase activity reached their maximum (92 and 89 U/g dry cell, *p*-nitrophenol-caprate used as substrate, assayed at 37°C, pH8.0) after induced for 96 h, while the lipase LipB52 displayed on *Saccharomyces cerevisiae *EBY100-pLHJ026 reached its maximum (92 U/g dry cell, assayed under the same condition) after induced for 48 h (Fig. 2) and the cells were harvested for further analysis.

### Localization of the displayed lipase LipB52

**Figure 3:**
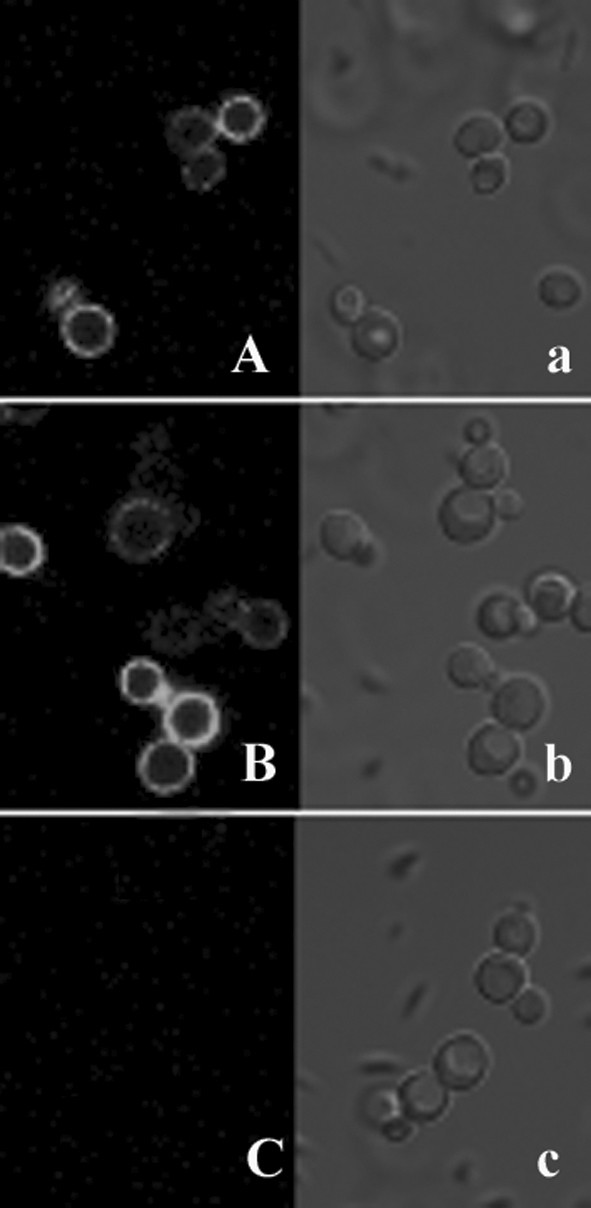
**Detection of the recombinant *Pichia pastoris *with immunofluorescence**. Fluorescence micrographs (panels A, B, and C) and differential interference contrast micrographs (panels a, b, and c) are shown. Panels A and a, *Pichia pastoris *KM71-pLHJ047; panels B and b, *Pichia pastoris *KM71-p LHJ048; panels C and c, *Pichia pastoris *KM71-pPIC9K (control).

Immunofluorescent labeling of cells was performed as described in Methods. The rabbit polyclonal anti-LipB52 antiserum was used as the primary antibody and the FITC-conjugated goat anti-rabbit immunoglobulin G was used as the secondary antibody. The green fluorescence outlining the immunostained LipB52 cells were clearly observed by the confocal laser scanning microscopy (CLSM) on the recombinant KM71-pLHJ047 and KM71-pLHJ048, the *Pichia pastoris *KM71 cells harboring the control plasmid pPIC9k were not immunostained (Fig. 3). The results confirmed that the LipB52 were displayed on the *Pichia pastoris *KM71 cell surface.

### Characterization and comparison of the displayed lipases

**Figure 4:**
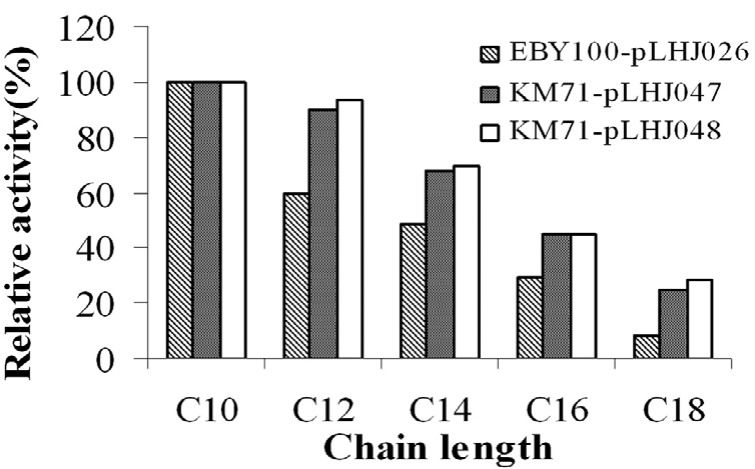
**The substrate specificity of the displayed lipases**. Lipase LipB52 displayed on *Pichia pastoris *and *Saccharomyces cerevisiae *exhibited evident hydrolysis activity towards the *p*-nitrophenol ester, but the optimum substrate was *p*-nitrophenol-caprate (C_10_).

**Figure 5:**
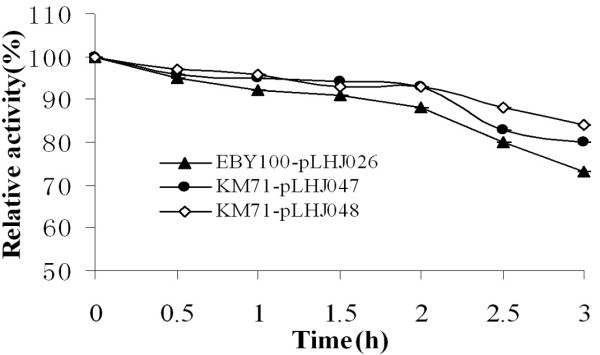
**The effect of temperature on stability of the displayed lipases**. The lipase LipB52 displayed on *Pichia pastoris *KM71-pLHJ047 and KM71-pLHJ048 cell surface retained over 90% activity after incubation at 60°C for 2 hours at pH 7.0, and they can still retained about 80% activity after incubation for 3 hours. The lipase LipB52 displayed on *Saccharomyces cerevisiae *EBY100-pLHJ026 retained near 90% activity after incubation at 60°C for 2 hours at pH 7.0 and it can only retained about 70% activity after incubation for 3 hours.

**Figure 6:**
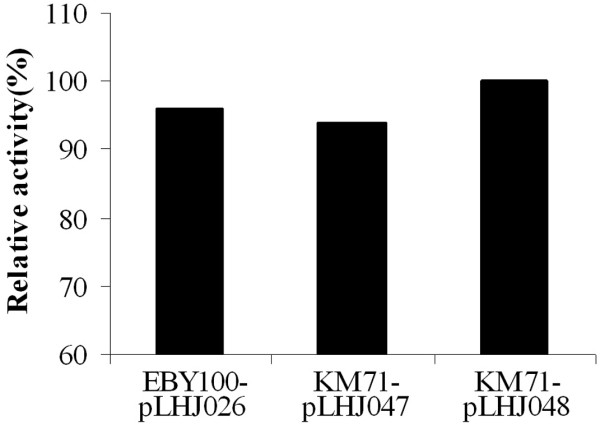
**Comparison on transesterification activity of the displayed lipases**. The displayed lipases exhibited similar transesterification activity.

The *p*-nitrophenol ester with carbon chain length ranging from C_10 _to C_18 _(C_10_, C_12_, C_14_, C_16_, C_18_) was used as the substrate to characterize the displayed lipase LipB52 produced by KM71-pLHJ047 and KM71-pLHJ048. The lipase LipB52 displayed on *Pichia pastoris *KM71 cell surface exhibited evident hydrolysis activity towards the *p*-nitrophenol ester, but the optimum substrate was *p*-nitrophenol-caprate (C_10_). Lipase LipB52 displayed on *Pichia pastoris *and *Saccharomyces cerevisiae *exhibited similar substrate specificity (Fig. 4). The lipase LipB52 displayed on *Pichia pastoris *KM71-pLHJ047 and KM71-pLHJ048 cell surface have a temperature optimum of 40°C at pH8.0. The hydrolysis activity reached 94 and 91 U/g dry cell respectively (assayed under their optimum condition). The lipase LipB52 displayed on *Saccharomyces cerevisiae *EBY100-pLHJ026 cell surface had a temperature optimum of 37°C at pH8.0, its maximum activity was 92 U/g dry cell. The lipase LipB52 displayed on *Pichia pastoris *KM71-pLHJ047 and KM71-pLHJ048 cell surface retained over 90% activity after incubation at 60°C for 2 hours at pH 7.0, and they can still retained about 80% activity after incubation for 3 hours. They exhibited better stability than the lipase LipB52 displayed on *Saccharomyces cerevisiae *EBY100-pLHJ026 cell surface (Fig. 5). The transesterification activity of the displayed lipases had no evident difference (Fig. 6).

## Discussion

Many reports indicated that the active site of *Pseudomonas *lipase was located at the C-terminal region [17-20], the lipase displayed on *Saccharomyces cerevisiae *cell surface by its C- terminus was fused to the cell-wall-anchored protein, α-agglutinin, exhibited no activity towards olive oil and a very low enzymatic activity towards *p*-nitrophenol butyrate [21]. The FLO protein is thought to produce cell adhesion via noncovalent interaction of its flocculation functional domain with the mannan chain of the cell wall. Its C-terminus was used to fuse with the N-terminus of target protein, it was successfully used to display active lipase based on *Saccharomyces cerevisiae *[8]. Considering the similarity between the cell walls of *Saccharomyces cerevisiae *and *Pichia pastoris *and the adhesive ability of the FLO protein, we attempted to utilize the relevant domain in a surface display system based on *Pichia pastoris*.

We successfully developed a *Pichia pastoris *surface display system utilizing flocculation functional domain of FLO protein, and had the lipase LipB52 expressed and displayed on *Pichia pastoris *KM71 cell surface. In the present study, two recombinant plasmids (pLHJ047 and pLHJ048) with the same components except the secretion signal sequences were constructed. The secretion signal sequence in pLHJ048 is the α-factor secretion signal sequence, while the secretion signal sequence in pLHJ047 is the one from *FLO*. As shown in the results, the signal sequence of *FLO *exhibits similar effect to α-factor signal sequence in *Pichia pastoris*. This *Pichia pastoris *surface display system is also expected to be effective for N-terminal immobilization of target proteins whose catalytic site is near its C-terminus.

The lipase LipB52 displayed on *Pichia pastoris *KM71 cell surface exhibited some similar biochemical characteristics to that displayed on *Saccharomyces cerevisiae *EBY100-pLHJ026 cell surface, but better stability. As shown in Tab. 1, the lipase gene was integrated into the genome when displayed on *Pichia pastoris *cell surface, it could be expressed steadily and can be fermented by using complete medium. However, the lipase gene was cloned in the episomal plasmid when displayed on *Saccharomyces cerevisiae *cell surface, it must be fermented by using minimal medium. Although the *Pichia pastoris *induction time was longer than *Saccharomyces cerevisiae*, the *Pichia pastoris *dry cell weight per liter (DCW/L) ferment culture was about 5 times than *Saccharomyces cerevisiae*. Further more the galactose used as the inducer for expressing lipase in *Saccharomyces cerevisiae *was more costly than the methanol used in *Pichia pastoris*. All these implied that the lipase displayed on *Pichia pastoris *cell surface is more suitable for being used as whole-cell biocatalysts than that displayed on *Saccharomyces cerevisiae *cell surface.

**Table 1:** Comparison of the properties of the recombinant *S. cerevisiae *and *P. pastoris*.

	Culture medium for expression	Promoter/Inducer	Location of the target gene	DCW/L^*a*^/Induction time
EBY100-pLHJ026	YNB-CAA containing glucose or galactose	*GAL1*/galactose	Cloned in the episomal plasmid	5.6 g/2 days
KM71-pLHJ047	BMGY, BMMY	*AOX1*/methanol	Integrated into genome	26.3 g/4 days
KM71-pLHJ048	BMGY, BMMY	*AOX1*/methanol	Integrated into genome	25.6 g/4 days

^*a *^DCW/L, Dry Cell Weight per liter ferment culture.

## Conclusion

A *Pichia pastoris *surface display system utilizing flocculation functional domain of FLO protein was developed, and the activity lipase LipB52 was expressed and displayed on *Pichia pastoris *KM71 cell surface. The lipase displayed on *Pichia pastoris *cell surface was more suitable for being used as whole-cell biocatalysts than that displayed on *Saccharomyces cerevisiae *cell surface.

## Methods

### Strains, vectors, culture media and enzymes

*Escherichia coli *DH5α, used as the recipient strain for recombinant plasmids, was grown in LB medium (1% tryptone, 0.5% yeast extract, 1%NaCl, pH7.0) at 37°C. The *FLO *gene was cloned from *Saccharomyces cerevisiae *ATCC 60715 (*MAT*α *FLO8 his4 leu2 STA1*). The recombinant *Saccharomyces cerevisiae *EBY100-pLHJ026 was constructed previously, the active lipase LipB52 was displayed on its cell surface. Yeast was grown in complete medium (YPD: 1% yeast extract, 2% peptone, 2% glucose) or selective medium (MD: 1.34% yeast nitrogen base, 4 × 10^-5 ^% biotin, 2% dextrose, 2% agar). BMGY (1% yeast extract, 2% peptone, 100 mM potassium phosphate, pH 6.0, 1.34% YNB, 4 × 10^-5 ^% biotin, 1% glycerol) and BMMY (1% yeast extract, 2% peptone, 100 mM potassium phosphate, pH 6.0, 1.34% YNB, 4 × 10^-5 ^% biotin, 0.5% methanol) were used for the recombinant *Pichia pastoris*. The *Pichia pastoris *KM71 and pPIC9K were purchased from Invitrogen Corporation. *Saccharomyces cerevisiae *EBY100-pLHJ026 (recombinant *Saccharomyces cerevisiae *displayed lipase LipB52, stored in our laboratory) were grown in YNB-CAA medium (0.67% yeast nitrogen base, 0.5% casamino acids) containing 2% glucose or galactose. The *exTaq *DNA polymerase, restriction enzymes, T4 DNA ligase and modification enzymes were purchased from TaKaRa Biotechnology (Dalian, China) Co., Ltd.

### Nucleic acid manipulation

DNA was purified and manipulated essentially as described by Sambrook et al [22]. DNA was sequenced with the dideoxy chain-termination method by an ABI 3730 automated sequencer from both strands by Invitrogen Biotechnology (Shanghai, China).

### Construction of the *FLO-lipB52 *surface expression vectors for *Pichia pastoris*

To amplify the *FLO *gene from *Saccharomyces cerevisiae *ATCC 60715 chromosomal DNA, the following two oligonucleotides were used as primers: FLOf-*Bgl *II (5'-acat*agatct*tatgacaatgcctcatcgctatatgttttt-3') and FLOr-*Eco*RI (5'-gat*gaattc*ggtgatttgtcctgaagatgatgatgacaaa-3'). Primers LipB52Pf-*Eco*R I (5'-aaa*gaattc*ccaacaaaaagagaggcaacagcaatg-3) and LipB52Pr-*Not *I (5'-aaa*gcggccgc*tccctccccacccttgtcgtcagg-3') were synthesized based on the sequence of *lipB52 *(GenBank accession number AY623009) and used to amplify *lipB52 *gene from the plasmid pLHJ018 containing *lipB52 *[7]. The PCR was performed as following: 95°C 10 min, followed by 30 cycles of amplification (95°C 1 min, 58°C 45 sec and 72°C 1 min) and 72°C 10 min after that. The purified PCR products, *FLO *and *lipB52 *gene, were digested with *Eco*R I, respectively, and ligated with T4 DNA ligase. Then the ligation product was used as the template to amplify the *FLO*-*lipB52 *fusion gene with primers FLOf-*Bgl *II and LipB52Pr-*Not *I. The *FLO*-*lipB52 *PCR product was purified and divided into two parts. One was digested with *Bgl *II and blunted with T4 DNA polymerase, then digested with *Not *I and ligated with pPIC9K digested with *Sna*B I and *Not *I, which reserved the α-factor secretion signal sequence. The other part was digested with *Bgl *II and *Not *I, and ligated with pPIC9K digested with *Bam*H I (*Bgl *II and *Bam*H I have the same cohesive end) and *Not *I, which discarded the α-factor secretion signal sequence. The recombinants were screened and identified with restriction enzymes and sequencing.

### *Pichia pastoris *transformation and expression

Electrocompetent cells of *Pichia pastoris *KM71 were prepared according to the supplier's instruction [23]. 10 μg recombinant plasmid linearized with *Sal *I was mixed with 80 μl electrocompetent cells, and electroporated by means of pulse discharge (1,500 V, 25 μF, Bio-Rad Gene Pulser) for 5 ms. After pulsing, 1 ml ice-cold 1 M sorbitol was immediately added to the cuvette. Then, 200 μl aliquots were spread on MD plates, and the plates were incubated at 30°C to screen for His^+ ^transformants according to their capacity to grow in the absence of histidine.

To detect the displayed lipase activity, the following manipulation was performed. His^+ ^clones were grown on BMGY plates at 30°C over night, and then transferred onto BMMY plates supplemented with 1% olive oil and 0.002% rhodamine B and induced at 30°C. 150 μl fresh methanol were added in the lid of plates every 24 h to induce the lipase protein expression. The recombinant strains with displayed active lipases were screened by BMMY plates supplemented with olive oil as substrate and rhodamine B as indicator. The recombinant strains were also identified by PCR with primers LipB52Pf-*Eco*R I and LipB52Pr-*Not *I.

Scale-up of expression was performed according to the supplier's instruction [23]. The recombinant strains were grown in 100 ml BMGY medium at 30°C, 250 rpm until the culture reached OD_600 _= 2.0–6.0. The cells were harvested by centrifugation and resuspended at a five-fold concentration in 20 ml BMMY medium to induce protein expression. The cells were incubated for 6 days at 30°C, 250 rpm, and fresh methanol was added to a concentration of 0.5% to maintain induction every 24 h. The cells were harvested by centrifugation for activity assay after washed with PBS (10 mM potassium phosphate buffer, pH 7.2, containing 150 mM sodium chloride). The harvested cells were immediately stored at -20°C over night and then quickly transferred to the lyophilizer (Freezon12, Labconco, USA) for 48 hours.

### Detection and characterization of the displayed lipase

Immunostaining was performed as follows. The rabbit polyclonal anti-LipB52 antiserum was raised against the recombinant LipB52 produced by *Pichia pastoris *KM71-pLHJ018 [7] and used as the primary antibody. The incubated yeast cells were washed with PBS. The cells were incubated with the primary antibody (1:50 diluted with 2% bovine serum albumin) on ice for 30 min with occasional mixing. After the cells had been washed with PBS, the secondary antibody, fluorescein isothiocyanate (FITC)-conjugated goat anti-rabbit immunoglobulin G (Jackson ImmunoResearch Laboratories Inc. USA, 1:200 diluted with 2% bovine serum albumin), was added and allowed to react with the cells on ice for 30 min in dark. The cells were then washed with PBS and detected by the confocal laser scanning microscopy (Zeiss LSM 510 META).

The release of *p*-nitrophenol (*p-*NP) from *p-*NP -derivative substrates was measured as described[24,25]. One unit (U) of hydrolysis activity was defined as the amount of enzyme that released 1 μmol of *p-*NP per minute under the assay conditions. The transesterification activity was assayed by gas chromatography as described [26].

### Expression of lipase LipB52 displayed on *Saccharomyces cerevisiae *EBY100-pLHJ026

Inoculate a single EBY100-pLHJ026 colony into 10 ml YNB-CAA containing 2% glucose and grow overnight at 30°C with shaking. After the OD_600 _= 2–5, centrifuge the cell culture at 8,000 rpm for 5 min at room temperature. Then resuspend the cell pellet in YNB-CAA medium containing 2% galactose to an OD_600 _of 0.5 to 1. Immediately remove a volume of cells equivalent to 2 OD_600 _units (e.g. OD_600 _of 0.5, remove 4 ml) and place on ice. This was used as zero time point. Incubate the cell culture at 20°C with shaking. Assay the cell culture every 12 h to determine the optimal induction time for maximum display. For each time point, read the OD_600 _and remove a volume of cells that is equivalent to 2 OD_600 _units. After the maximum display, the cells were harvested by centrifugation for activity assay after washed with PBS and lyophilized.

## Authors' contributions

ZJ carried out construction of vectors, the sequence alignment and drafted the manuscript. RR carried out *Pichia pastoris *transformation and expression, participated in the immunofluorescent labeling. BG carried out detection and characterization of the displayed lipase. XT participated in the immunofluorescent labeling. YM performed the statistical analysis. DW participated in the design of the study and coordination and helped to draft the manuscript. All authors read and approved the final manuscript.
